# Expression of Concern: Novel Schiff base Mn(iii) and Co(ii) complexes supported on Co nanoparticles: efficient and recyclable magnetic nanocatalysts for alcohol oxidation

**DOI:** 10.1039/d4ra90113d

**Published:** 2024-09-30

**Authors:** Hassan Keypour, Shokoufeh Ghahri Saremi, Hojat Veisi, Reza Azadbakht

**Affiliations:** a Faculty of Chemistry, Bu-Ali Sina University Hamedan 65174 Iran haskey1@yahoo.com; b Department of Chemistry, Payame Noor University Tehran Iran

## Abstract

Expression of Concern for ‘Novel Schiff base Mn(iii) and Co(ii) complexes supported on Co nanoparticles: efficient and recyclable magnetic nanocatalysts for alcohol oxidation’ by Hassan Keypour *et al.*, *RSC Adv.*, 2016, **6**, 77020–77029, https://doi.org/10.1039/C6RA15967B.

*RSC Advances* is publishing this expression of concern in order to alert readers that concerns have been raised over the integrity of the data published in this article. The authors have been contacted but have not responded to requests to provide raw data. An expression of concern will continue to be associated with the article until a conclusive outcome is reached.

Signed: Laura Fisher

Date: 18th September 2024

Executive Editor, *RSC Advances*

